# Defect-Driven Selectivity Inversion in ZnO Gas Sensors via Y^3+^ and Dy^3+^ Doping for Enhanced VOC Detection with a Suppressed NO_2_ Response

**DOI:** 10.3390/s26154675

**Published:** 2026-07-23

**Authors:** Imen Massoudi, Driss Lahem, Marc Debliquy, Norah Abdullah Algarou, Reem Khalid Aldakheel, Norah Alonizan, Amor Ben Ali, Muhammad Younas, Bayan Almohsen, Talal F. Qahtan, Fatemah M. Barakat

**Affiliations:** 1 Department of Physics, College of Science, Imam Abdulrahman Bin Faisal University, P.O. Box 1982, Dammam 31441, Saudi Arabia; 2 Basic & Applied Scientific Research Center, Imam Abdulrahman Bin Faisal University, P.O. Box 1982, Dammam 31441, Saudi Arabia; 3 Materia Nova, Materials R&D Centre, Parc Initialis, Avenue Nicolas Copernic 3, 7000 Mons, Belgium; 4 Materials Science Department, University of Mons, 7000 Mons, Belgium; 5 Department of Chemistry, College of Science, Imam Abdulrahman Bin Faisal University, P.O. Box 1982, Dammam 31441, Saudi Arabia; 6 Core Research Facilities (CRF), King Fahd University of Petroleum and Minerals (KFUPM), Dhahran 31261, Saudi Arabia; 7 Physics Department, College of Science and Humanities in Al-Kharj, Prince Sattam Bin Abdulaziz University, Al-Kharj 11942, Saudi Arabia; 8 Department of Physics and Astronomy, College of Science, King Saud University, Riyadh 11451, Saudi Arabia

**Keywords:** ZnO semiconductors, gas sensors, trivalent RE ions, defect engineering, selectivity inversion, NO_2_ suppression, VOC detection

## Abstract

In this work, we demonstrate a defect-mediated strategy to fundamentally reprogram the selectivity of ZnO gas sensors through targeted doping with two types of trivalent rare-earth ions. We incorporate yttrium (Y^3+^) and dysprosium (Dy^3+^) into the ZnO host via a high-energy ball-milling (HEBM)-assisted solid-state reaction. On the basis of structural, optical, and spectroscopic characterizations, we demonstrate a significant increase in the oxygen vacancy density and a reduction in the band gap energy. The gas sensing tests revealed a remarkable inversion of selectivity: while doping suppressed the NO_2_ response by 86% to 94%, it increased the sensitivity to VOCs. Dy doping produced a selective sensor for ethanol (S = 9.61, 5.62 × selectivity over NO_2_), and Y doping produced a selective sensor for acetone (S = 8.27, 2 × selectivity over NO_2_). This dopant-specific defect engineering provides a direct pathway to adapt ZnO sensors to the selective detection of VOCs in environmental monitoring.

## 1. Introduction

A variety of platforms are available for gas sensors, including catalytic sensors, infrared sensors, photoionization detectors (PIDs), acoustic sensors, and metal–insulator–metal sensors (MIMs) [1,2]. In parallel, other categories of sensors based on optical phenomena, such as photoluminescence, have emerged as a promising alternative, offering high selectivity thanks to gas-modulated light emission changes [3]. Among these technologies are metal–oxide–semiconductor (MOS)-based sensors, which are widely employed in environmental monitoring [4,5]. A key characteristic of MOS sensors is their chemiresistive response, where the electrical resistance changes in response to variations in the surrounding chemical environment [6].

Zinc oxide (ZnO) is an n-type semiconductor that is promising for the detection of chemoresistive gases [7]. It has a wurtzite crystal structure, a wide band gap energy (3.37 eV), high electron mobility, and tunable surface chemistry that make it particularly suitable for the detection of a wide range of gases, including volatile organic compounds (VOCs), such as alcohols (C_2_H_5_OH) and acetone (CH_3_)_2_CO, and nitrogen oxides such as NO_2_ [8,9]. In addition, ZnO is more affordable than many other detection materials and is compatible with scalable synthesis and microfabrication pathways, meeting the main challenges in terms of cost, stability, and manufacturability [10,11,12,13].

**Figure 1:**
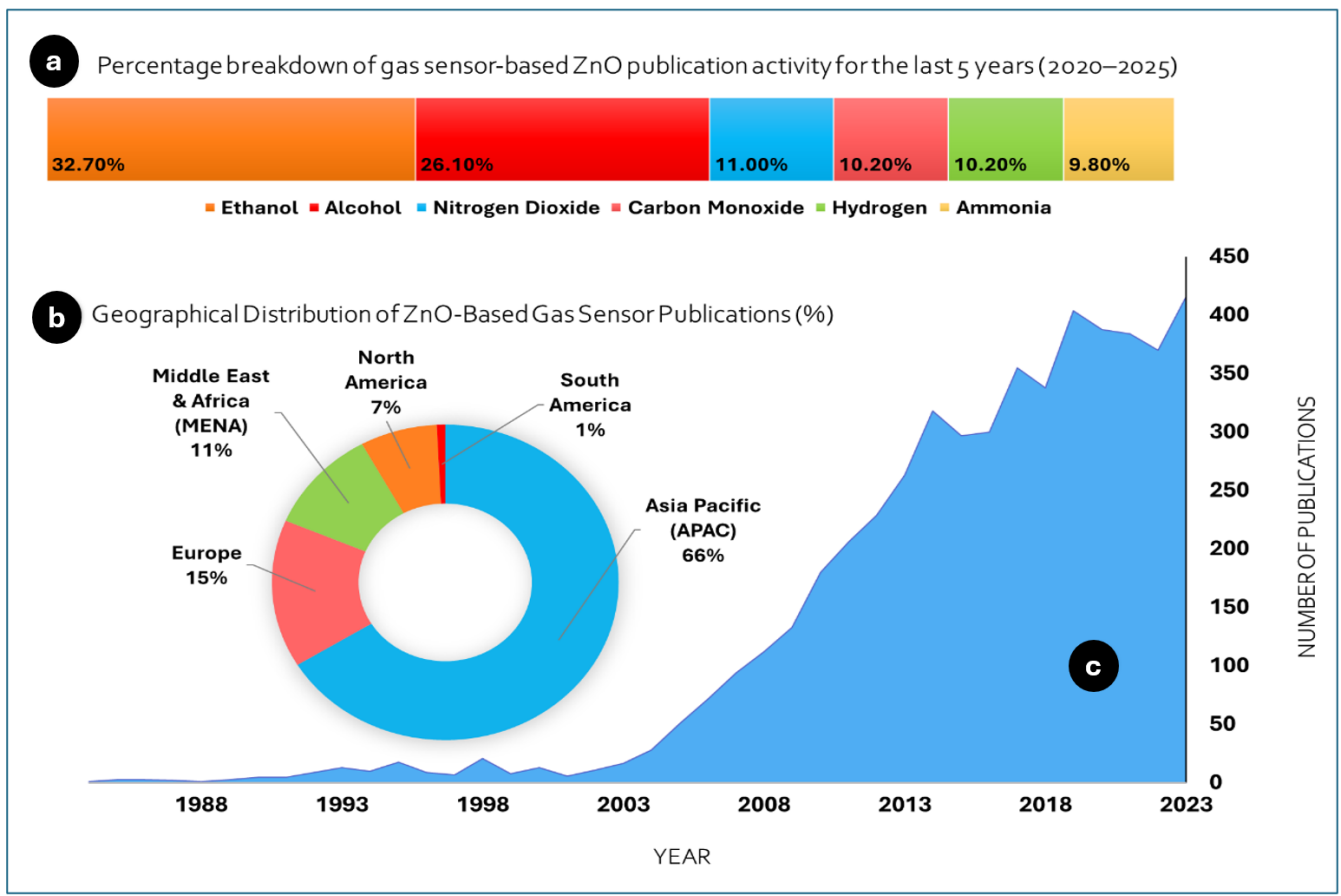
Global trends in ZnO-based gas sensor research. (**a**) Percentage distribution of sensor activity by target gas. Ethanol and alcohol collectively represent 58% of recent studies (2020–2024). (**b**) Regional distribution of publications, highlighting the predominance of the Asia-Pacific region (APAC) (66%). (**c**) Volume of publications by year (1988–2024). Data from Scopus using the keywords “zinc oxide” and “gas sensor”.

Figure 1 illustrates recent trends in publications and the regional contributions of research on ZnO-based gas sensors. As shown, ethanol and alcohol have dominated sensor activity over the past four years, highlighting their importance in the detection of VOCs [4,5,6,14]. However, ZnO faces limitations in gas sensor applications, particularly its high operating temperature (OT) and its low selectivity [15,16]. To address these challenges, several approaches have been explored, including nanostructuring, surface functionalization, heat treatment, and doping. [2,17,18]. Among these, the doping technique has shown an improvement in the reactivity of the ZnO-based gas sensor by modulating the surface electronic structure [19] and the creation of active sites [20,21,22].

Rare-earth (RE) elements are particularly promising dopants. They improve sensors’ efficiency, especially selectivity and sensitivity, due to fast oxygen ion mobility and unique electronic structures [22,23,24,25]. Yttrium (Y) and dysprosium (Dy), heavy RE oxides, have attracted increasing interest in the modification of ZnO [26,27]. Recent studies show that their incorporation improves the gas detection performance [25,28,29,30]. Table 1 presents a literature review scan of the RE dopants that have been used to improve ZnO gas sensors either for lowering the OTs and/or broadening the gas detection selectivity and capabilities. Although Dy and Y are among the most investigated RE dopants for ZnO gas sensors, previous studies have typically examined their effects individually on gas sensitivity, leaving a systematic comparison of their ability to reverse selectivity between oxidizing and reducing gases largely unexplored [31]. For instance, Y-doped ZnO nanofibers prepared by electrospinning have been shown to exhibit a high response and good selectivity toward acetone, demonstrating the potential of Y doping for VOC detection [32].

## 2. Materials, Methods, and Characterizations

### 2.1. Materials and Synthesis

**Figure 2:**
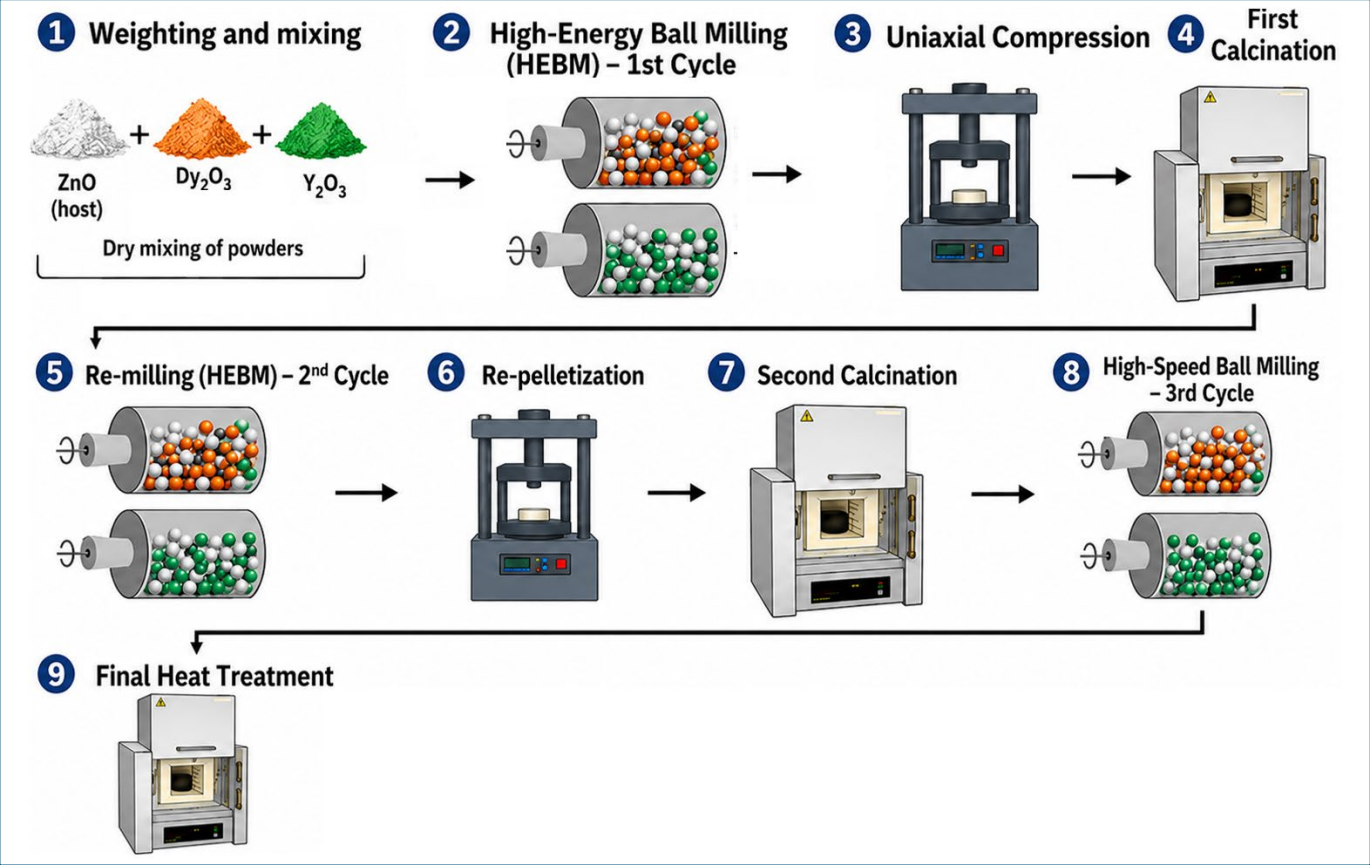
Schematic of Dy- and Y-doped ZnO nanoparticle synthesis via HEBM-assisted solid-state reactions.

In this study, analytical grade ZnO (Sigma-Aldrich, Merck KGaA, Darmstadt, Germany, ≥99.9%) served as the host material, while Dy_2_O_3_ and Y_2_O_3_ (both Sigma-Aldrich, Merck KGaA, Darmstadt, Germany, ≥99.9%) were used as dopant sources for the Dy^3+^ and Y^3+^ ions, respectively. To balance an efficient substitution of the lattice with a minimum secondary phase formation, a nominal doping level of 4 wt.% was selected for each RE element. The resulting compositions, denoted ZY4 (ZnO + 4 wt.% Y_2_O_3_) and ZD4 (ZnO + 4 wt.% Dy_2_O_3_), were prepared via a solid-state reaction assisted by high-energy bal- milling (HEBM), an evolutionary solvent-free route that promotes a homogeneous distribution of dopants and gives nanostructured powders that are well adapted to gas detection applications (Figure 2). We selected the nominal doping level of 4 wt.% on the basis of three considerations: (i) preliminary screening experiments conducted at increasing dopant concentrations, which identified 4 wt.% as the optimal balance between structural modification and secondary phase suppression; (ii) previous literature reports on RE-doped ZnO gas sensors, where similar concentrations (3–5 wt.%) yielded optimal sensing performance [25,28,29,30]; and (iii) the need to ensure sufficient substitutional doping while minimizing the formation of inactive secondary oxide phases, which would dilute the active surface sites.

In a typical synthesis, we manually pre-mixed the stoichiometric amounts of precursors. Then, we subjected the powder to high-energy ball-milling (Emax, Retsch, Germany) using zirconia balls (5 mm diameter) with a ball-to-powder mass ratio of 15:1. The milling process was performed at 1000 rpm for 2 h with periodic 15 min pauses to avoid overheating. The milled powder was compacted into pellets (50 MPa, 15 min) and calcined in air at 1000 °C for 8 h (10 °C min^−1^) to drive the solid-state reaction and initial phase formation. To further enhance the homogeneity and crystallinity, the materials were re-milled, re-pelletized, and subjected to a third identical calcination. After a final milling step, the powder was sintered at 500 °C for 5 h in air to stabilize the nanostructure and activate surface properties. The three-step grinding and annealing process was used to ensure the complete solid-state reaction and homogeneity of the final product.

### 2.2. Gas Sensor Fabrication and Testing

#### 2.2.1. Sensor Paste Preparation and Deposition

**Figure 3:**
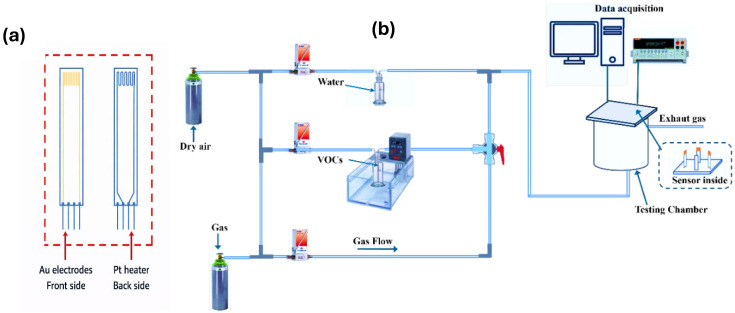
(**a**) A schematic of the alumina substrate with interdigitated gold electrodes and a Pt heater. (**b**) The schematic of the custom gas sensing measurement setup.

We prepared the detection paste by carefully mixing each synthesized powder with terpineol (60 wt.%) to obtain a homogeneous consistency paste. We then deposited the obtained paste on alumina substrates (Al_2_O_3_, C-MAC Micro Technology, Ronse, Belgium) using the screen-printing technique. Each one of the substrates was equipped with interdigitated gold electrodes and an integrated platinum (Pt) heating element for temperature control (Figure 3a). The interdigitated gold electrodes had an inter-arm spacing of 250 µm, with each arm measuring 4 mm in length. The integrated Pt heater consisted of a serpentine resistor (4 arms of 5 mm each, spaced 1 mm apart) powered by a precision DC supply. The operating temperature was controlled by applying electrical power to this heating element, following prior calibration correlating applied power to the resulting sensor temperature. To ensure the film’s stability and adhesion, the printed sensors were thermally treated at 400 °C for 2 h in a muffle furnace. The film’s thickness was approximately 45 µm, as determined by profilometry (Dektak XT, Bruker Corporation, Billerica, MA, USA). For each composition, three sensor devices were fabricated. The devices selected for testing were chosen for comparable film thickness and heater resistance, reducing device-to-device variability.

#### 2.2.2. Gas Sensing Measurement System

The gas detection evaluations were carried out using a tailor-made measurement system (Figure 3b). The system consisted of a Teflon test chamber (8 cm in diameter and 9 cm in height) housing the sensor, which was connected to a data acquisition unit for real-time resistance monitoring. The OT of the sensors was precisely controlled via the integrated Pt heater. The target gases (NO_2_, ethanol, or acetone) were introduced into the chamber using calibrated mass flow regulators, while a separate airflow was used for basic recovery and humidity control. The relative humidity (RH) was maintained at 50 ± 5% by bubbling the carrier gas through distilled water. The total gas flow rate was maintained at 1000 sccm using calibrated mass flow meters (Bronkhorst High-Tech BV, Ruurlo, The Netherlands). Ethanol and acetone vapors were generated using a bubbler filled with an aqueous solution of the target VOC. The bubbler solution was maintained at 25 °C in a water bath, and the actual VOC concentration and humidity level were verified using a commercial TVOC meter (GrayWolf Sensing Solutions, Shelton, CT, USA). The sensor’s resistance was measured using a bridge circuit with a load resistor (R_L_) in series with the sensor, with the supply voltage selected to ensure that measurements were within the linear response range (U_S_ < 1 V).

Before each measurement, we stabilized the sensor in synthetic air until we reached a stable base resistance. The gas exposure cycle consisted of a 5 to 10 min injection of the target gas, followed by a 10 to 20 min recovery period in the air. All dynamic measurements were carried out at the optimum OT found for each gas–sensor sample. For each composition, three sensor devices were fabricated. The devices selected for testing were chosen on the basis of comparable film thickness and heater resistance values, which reduced device-to-device variability. The data presented in this work correspond to representative measurements from the selected devices.

### 2.3. Characterizations

We characterized the synthesized samples using a wide range of techniques to analyze their structural, morphological, optical, and functional properties. The structural analysis was carried out using X-ray powder diffraction (XRD) on an MPD PRO Panalytical diffractometer (Malvern Panalytical Ltd., Almelo, The Netherlands) equipped with Cu K*α* radiation (λ = 1.54 Å), operating in Bragg–Brentano geometry. The data were collected over a range of 2θ from 20° to 80° with a step size of 0.02° and 30 s per step to identify the crystallographic phases and estimate the lattice parameters. Fourier transform infrared spectroscopy (FTIR) was carried out with a Shimadzu IRAffinity-1S spectrometer (Shimadzu Corporation, Kyoto, Japan) in the range of 400–4000 cm^−1^ mixed with KBr pellets to identify the functional groups and the binding characteristics in the studied samples. The surface morphology and the microstructure of the samples were examined by FEI Quanta 600 scanning electron microscopy (SEM) (Thermo Fisher Scientific, Hillsboro, OR, USA), while the elemental distribution and the chemical composition were confirmed by energy dispersive X-ray spectroscopy (EDX). The surface chemistry and elemental composition were studied using X-ray photoelectron spectroscopy (XPS) with a K-Alpha^+^ system (Thermo Fisher Scientific, East Grinstead, UK). This system provided us with a high-resolution spectrum under an ultra-vacuum and allowed the characterization of the oxidation states and surface defects such as oxygen vacancies (OVs). The optical properties were evaluated by UV–visible diffuse reflectance spectroscopy (DRS) in the wavelength range from 200 to 900 nm using a SolidSpec-3700-spectrometer (Shimadzu Corporation, Kyoto, Japan) to evaluate the light absorption behavior and estimate the band gap energy. The photoluminescence (PL) spectra were recorded at ambient temperature using a Cary Eclipse spectrofluorometer EL08083851 (Agilent Technologies, Santa Clara, CA, USA) equipped with a xenon arc lamp, operating in the range of 350 to 600 nm, to study the recombination behavior and the emission related to defects.

## 3. Results and Discussion

### 3.1. Crystal Structure and Phase Analysis (XRD)

**Figure 4:**
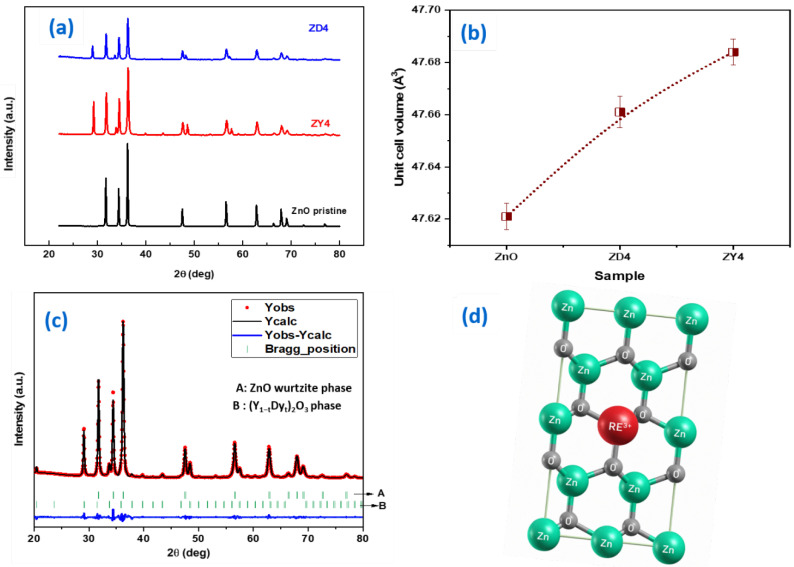
(**a**) XRD patterns of ZnO, Y-doped ZnO (ZY4), and Dy-doped ZnO (ZD4) powders; (**b**) unit cell volume of pristine ZnO, ZY4, and ZD4; (**c**) Representative Rietveld refinement plot for ZD4 showing observed (red circles), calculated (black line), and difference (blue line) profiles, with Bragg peak positions for the ZnO wurtzite phase (upper ticks, labeled ‘A’) and the secondary (Y_1−t_Dy_t_)_2_O_3_, phase (lower ticks, labeled ‘B’). (**d**) Schematic representation of the ZnO wurtzite crystal structure showing Zn^2+^ sites (green), O^2−^ sites (grey), and substitution of Zn^2+^ by RE^3+^ ions (red).

We analyzed the crystalline structure of the studied samples by the X-ray diffraction (XRD) technique. The diffracted patterns for pure ZnO, Y-doped ZnO (ZY4), and Dy-doped ZnO (ZD4) are illustrated in Figure 4a. All the major diffraction peaks of the doped ZnO powders correspond to the hexagonal structure of the ZnO wurtzite (JCPDS n° 36-1451), confirming that doping had not altered the primary phase. However, a careful inspection reveals that the diffracted peaks of ZY4 and ZD4 are slightly offset towards the lower angles relative to the pure ZnO. This could indicate an expansion of the grating due to the incorporation of the dopant. To confirm this, we performed a quantitative analysis using the Rietveld refinement (Figure 4c). The refined unit cell parameters are summarized in Table 2. As expected, a clear expansion in the lattice parameters (a, c) and in the unit cell volume (ϑ) is observed for both the two doped samples (Figure 4b). This expansion of the lattice is a direct consequence of the substitution of the Zn^2+^ ions (an ionic radius of 60 pm in tetrahedral coordination) by the larger Y^3+^ (90 pm) or Dy^3+^ (91 pm) ions [41]. The reliability factors obtained from the Rietveld refinement (R_p_ = 3.2%, R_wp_ = 4.8%, χ^2^ = 1.9) confirm the accuracy of the phase quantification [42]. A schematic representation of the ZnO wurtzite crystal structure is shown in Figure 4d, illustrating the hexagonal unit cell with Zn^2+^ ions tetrahedrally coordinated by O^2-^ ions. The substitution of Zn^2+^ sites by larger RE^3+^ ions resulted in the observed lattice expansion.

**Figure 5:**
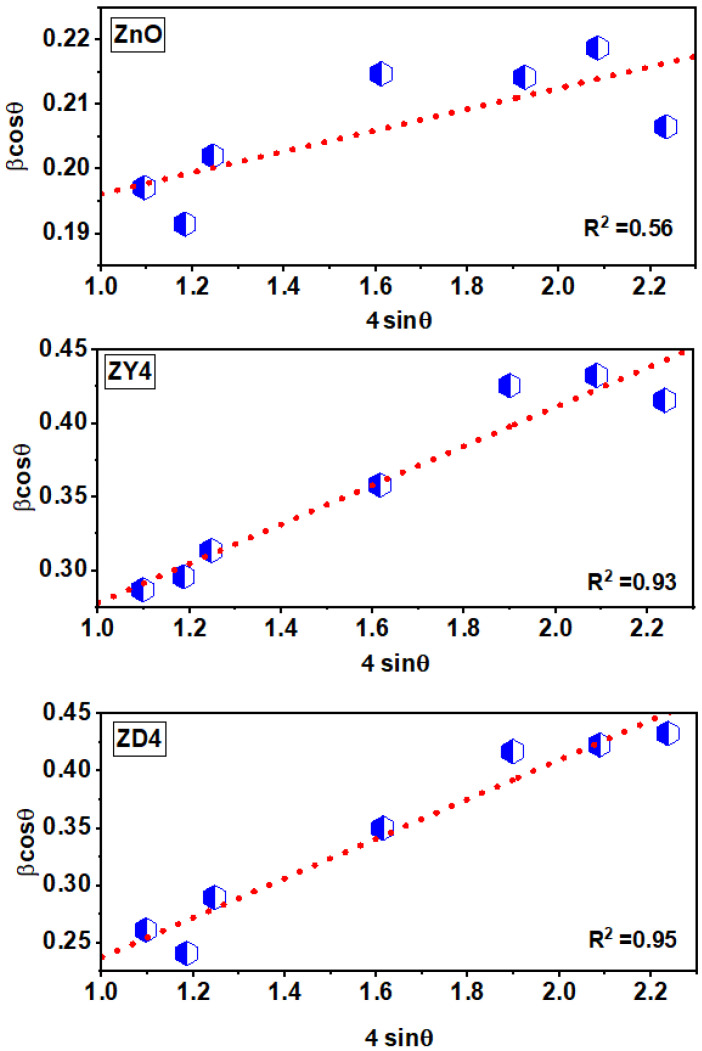
Williamson–Hall plots of pure, Y-, and Dy-doped ZnO powders, showing the linear fits of β cosθ vs. 4 sinθ used to determine the crystallite size from the intercept and the microstrain from the slope.

To deconvolve the effects of microstrain and crystallite size, we used the Williamson–Hall (W-H) analysis [43], based on the equation

(1) $$\beta \;\cos\theta =\frac{k\lambda }{D}+4\;\varepsilon \;\sin\theta \;$$

where β is the FWHM, θ stands for the Bragg angle, D is the crystallite size, k represents the shape factor (0.9), ε is the microstrain, and λ is the X-ray wavelength (1.5406 Å). As shown in Figure 5, a plot of βcosθ vs. 4sinθ yields a linear fit, where the intercept gives D and the slope gives ε. The plots for ZnO, ZY4, and ZD4 show positive slopes, indicating tensile strain. The two doped samples have significantly higher microstrain values than the pure ZnO, resulting from the distortion of the lattice induced by larger doping cations. In addition, the calculated crystallite size exhibited an increase from 7.7 nm for pure ZnO to 9.5 nm for ZY4 and 21.2 nm for ZD4 (Table 3). The pronounced growth of crystallites in ZD4 suggests that Dy doping may boost the crystals’ growth under the applied synthesis conditions, in comparison with Y.

As shown in Figure 5, the plots for ZnO, ZY4, and ZD4 show positive slopes, indicating tensile strain. The two doped samples have significantly higher microstrain values than the pure ZnO, resulting from the distortion of the lattice induced by larger doping cations. In addition, the calculated crystallite size exhibited an increase from 7.7 nm for pure ZnO to 9.5 nm for ZY4 and 21.2 nm for ZD4 (Table 3). The pronounced growth of crystallites in ZD4 suggests that Dy doping may boost the crystal growth under the applied synthesis conditions, in comparison with Y.

The refinement reveals a minor secondary phase, identified as cubic (Y1-tDyt)_2_O_3_, quantified at 11.9 wt% in ZY4 and 8.9 wt% in ZD4. Importantly, the dominant phase remains the ZnO wurtzite structure (>88 wt%), confirming that the majority of RE^3+^ ions were successfully incorporated into the ZnO lattice. The presence of this minor secondary phase is consistent with previous reports on mechanochemically synthesized doped ZnO systems [12,13,44].

### 3.2. Vibrational Spectroscopy (FTIR)

**Figure 6:**
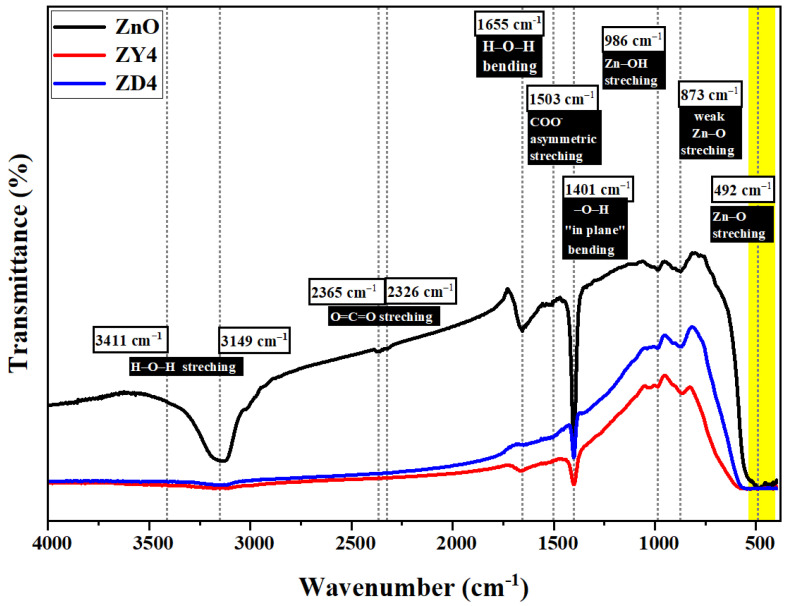
FTIR spectra of pure, Y- and Dy-doped ZnO powders. The region of interest around 500 cm^−1^ (highlighted in yellow) shows the Zn-O stretching peak.

To understand how RE doping (Dy^3+^ or Y^3+^) modifies the chemical environment and lattice dynamics of ZnO nanoparticles, Fourier transform infrared (FTIR) spectroscopy was recorded for all samples across the 400–4000 ${cm}^{-1}$ range, as shown in Figure 6. In the high-frequency region, a wide absorption band appears at 3411 and 3149 ${cm}^{-1}$. The O-H stretching bands observed at 3411 and 3149 cm^−1^ are attributed to surface-adsorbed water molecules [45]. We also observed the characteristic H-O-H bending vibration at 1655 ${cm}^{-1}$, along with the in-plane O-H bending mode at 1401 ${cm}^{-1}$ [46]. Moreover, the weak doublet peaks observed at 2365 and 2326 cm^−1^, assigned to asymmetric stretching of atmospheric CO_2_ [47], show a drop in intensity in doped samples. We can assign the weak carboxylate anion (COO^−^) asymmetric stretching modes observed at 1510 and 1562 ${cm}^{-1}$ and their obscured symmetric counterparts (recorded at 1367 cm^−1^) to minimal contributions from organic residues, emphasizing the dominance of lattice-related vibrations [48].

The low-wavenumber domain (less than 1000 cm^−1^) is considered to be the region with the most significant FTIR changes because of the dominance of the Zn-O bond vibrations. As one can see, the undoped ZnO exhibits a sharp Zn-O stretching peak at 492 cm^−1^, a signature of a well-ordered hexagonal wurtzite structure [49,50]. After doping, this peak becomes wide and shifts towards higher wavenumbers: 542 cm^−1^ for ZD4 and 559 cm^−1^ for ZY4. We can attribute the peak shift and broadening to the lattice distortion and the bond weakening induced by the substitution of Zn^2+^ ions (65.38 u) with larger, heavier Dy^3+^ (162.5 u) and Y^3+^ (8.9 u) ions [49,51]. Similar findings were also reported in the FTIR spectra of Dy^+3^- or Y^+3^-doped ZnO according to different ratios and synthesis techniques [52,53,54]. Weak Zn-OH (986 cm^−1^) and Zn-O (873 cm^−1^) vibrations further corroborate the structural deformation upon doping [49,50].

### 3.3. Morphology and Elemental Composition (SEM/EDS)

**Figure 7:**
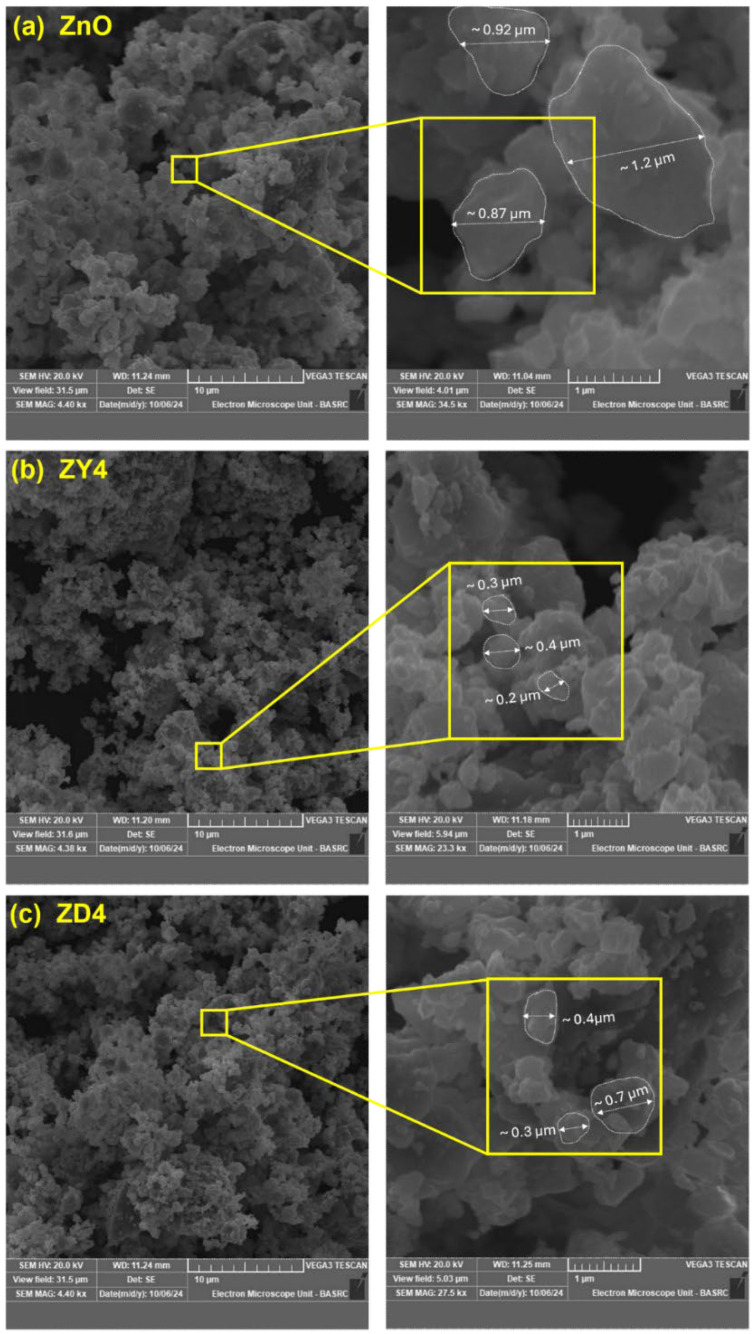
SEM morphological micrographs of (**a**) pristine ZnO, (**b**) Y-doped ZnO (ZY4), and (**c**) Dy-doped ZnO (ZD4) at low magnification (10 µm) and high magnification (1 µm), showing crystallite details. Dotted lines indicate three typical crystallites in each sample.

In Figure 7, an SEM micrograph of the ZnO is shown in comparison with its singly doped counterparts, at two different magnifications. As one can see, the pristine powder sample is composed of irregular and non-uniform grains ranging from about 0.5 to 2.0 µm in size, with significant agglomeration (Figure 7a). Upon Y doping (Figure 7b), the morphology becomes finer with more fragmented particles (~200–500 nm) distributed throughout the sample, with three typical particles indicated by dotted lines. The ZD4 powder (Figure 7c) exhibits a more aggregated porous structure with particles in the range of ~200–800 nm. The dopant ions appear to inhibit crystallite coalescence during calcination, resulting in smaller and more dispersed particles compared with the pristine ZnO.

**Figure 8:**
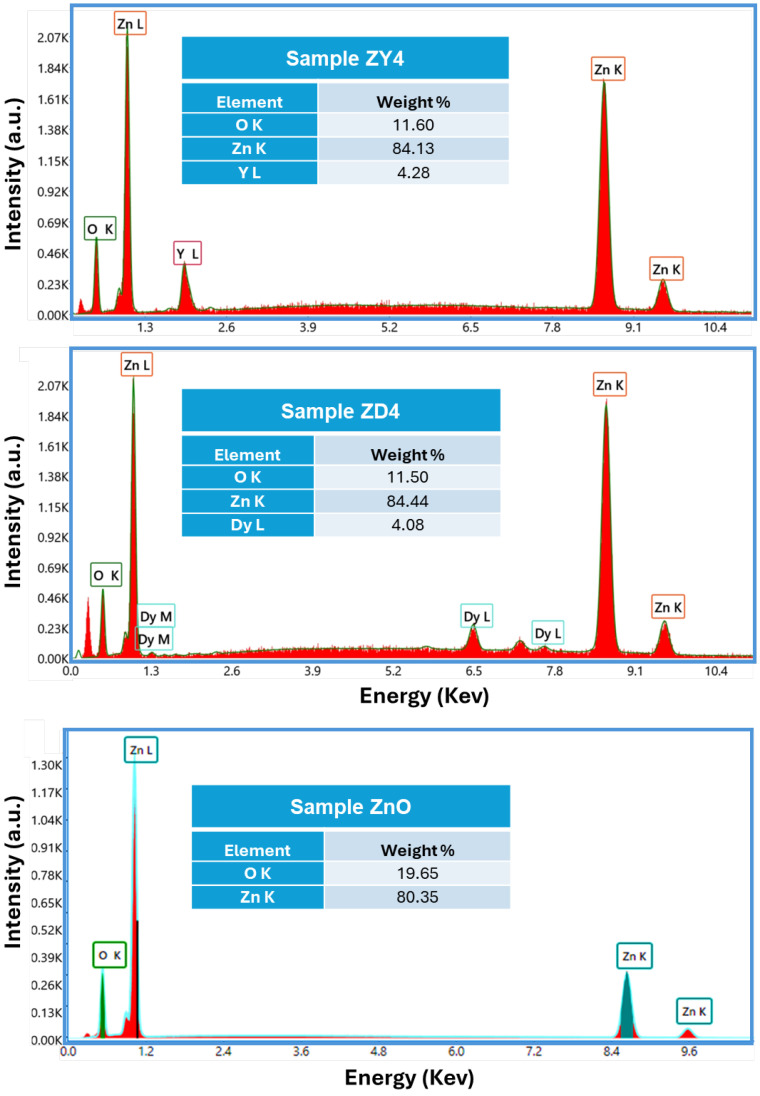
Energy dispersive X-ray patterns (EDS) of pure, Y-, and Dy-doped ZnO powders.

Figure 8 shows the EDS spectra of the corresponding compounds studied. EDS analysis, which provides elemental composition data, confirms the successful incorporation of dopant elements. Indeed, a quantitative analysis of ZY4 shows a distinct Y peak (4.28 wt.% Y), whereas ZD4 shows a distinct Dy peak (4.08 wt.% Dy). These weight percentages confirm the presence of the dopants and are consistent with the nominal addition of 4 wt.% Y_2_O_3_ or Dy_2_O_3_ precursors. The corresponding atomic percentage, which more directly reflects the substitutional doping level in the ZnO lattice, is lower due to the higher atomic mass of the rare-earth elements. Notably, the intensity of the oxygen K (O K) peak (about 0.60 keV) in the doped samples (ZY4, ZD4) exceeds that of the pristine ZnO sample (about 0.33 keV). The observed increase can be primarily associated with the generation of OVs, a common charge-compensation mechanism for trivalent substitution [55].

### 3.4. Surface Chemistry and Defect Analysis (XPS)

**Figure 9:**
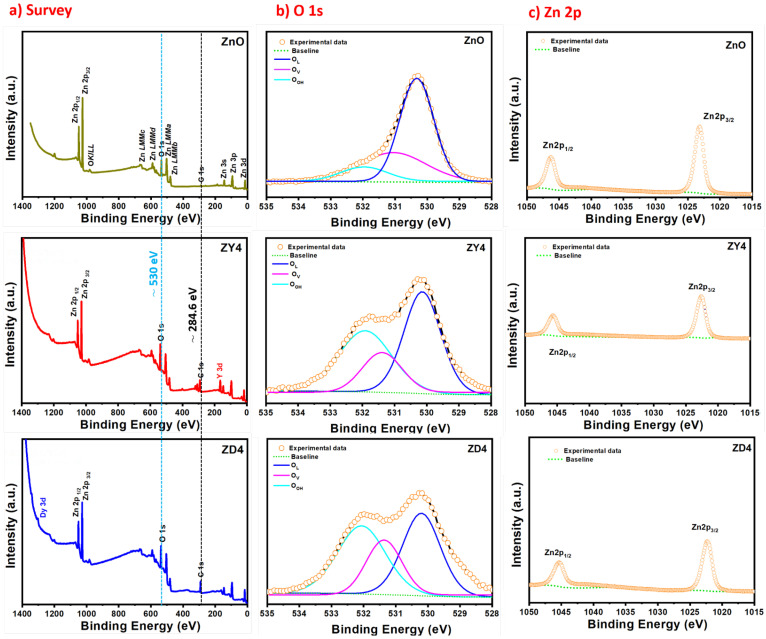
XPS spectra of undoped ZnO and singly doped ZY4 and ZD4. (**a**) Survey scans. (**b**) O 1s and (**c**) Zn 2p high-resolution spectra. The C 1s vertical guideline at 284.6 eV confirms consistent charge correction across all samples.

We present the XPS survey spectra of pristine and doped samples in Figure 9a. The spectrum of pure ZnO confirms the high purity: it demonstrates a characteristic peak for Zn 2p (1022, 1045 eV), O 1s (~530 eV), characteristics of the Zn LMM auger (980–1040 eV), and a minor peak of C 1s (285 eV) from adventitious carbon [56,57]. The successful incorporation of dopant elements is explicitly demonstrated by the emergence of distinct 3d Y peaks (158–162 eV) in the ZY4 spectrum, and 3d Dy peaks (1290–1330 eV) in the ZD4 spectrum, without any foreign or external elemental signatures [58,59]. The vertical guidelines in Figure 9a highlight the peak positions, and the C 1s vertical guideline at 284.6 eV confirms consistent charge correction across all samples. Figure 9b confirms the high-resolution O 1s spectra, which have been deconvoluted, as shown, into three Gaussian components to quantify the changes in the chemical environment of oxygen [56]. The most dominant peak at the lowest binding energy, which appears at ~530.3 eV, is attributed to the lattice oxygen (O_L_) linked to zinc in the wurtzite structure. The intermediate component at ~531.4 eV is attributed to the oxygen vacancies (O_V_) and defect-related oxygen species. The high binding energy peak located at ~532.0 eV is attributed to the surface hydroxyl groups (O_OH_) and adsorbed water. The lattice oxygen peak dominates in the pristine ZnO. However, doped samples with trivalent ions display a noticeable increase in the relative surface area of the peak associated with oxygen vacancies, which is a direct signature of the charge-compensation phenomenon. When we substitute a Zn^2+^ ion with larger trivalent cations such as Y^3+^ or Dy^3+^, a local positive charge imbalance is generated and directly neutralized by the formation of a negatively charged OV. The 2p Zn spectrum for all powder samples shown in Figure 9c exhibits the characteristic spin-orbit doublet 2p_3/2_ and 2p_1/2_, with a constant binding energy separation of 23 eV. This confirms that Zn maintains its +2-oxidation state. Nonetheless, a subtle shift toward lower binding energy is observed for the doped samples, indicating an increase in electron density around the zinc cations. For instance, we observe from the surveys (Figure 9a) that the 3d Y spectrum of ZY4 displays the distinctive doublet characteristic of Y^3+^ at 158.8 eV (3d_5/2_) and 160.8 eV (3d_3/2_). Similarly, the 3d Dy spectrum of ZD4 reveals a large and complex multiplet. It lies between 1295 and 1330 eV. That structure is a definitive imprint of Dy^3+^ in an oxide matrix. Consequently, the quantification of these defects reveals a clear hierarchy: ZD4 > ZY4 > ZnO.

### 3.5. Optical Properties: Band Structure and Defect States

#### 3.5.1. UV–Vis DRS

**Figure 10:**
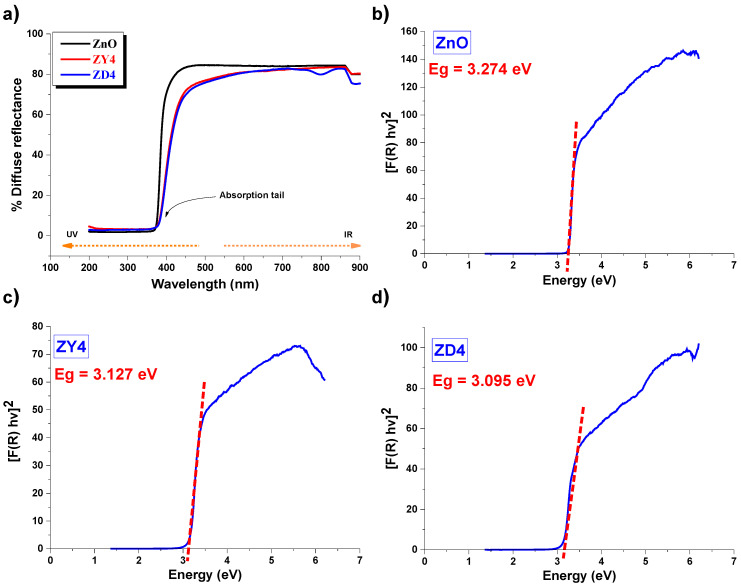
(**a**) DRS profiles and (**b**–**d**) K-M plots of pure, Y-doped (ZY4), and Dy-doped (ZD4) ZnO powders, respectively.

UV–vis DRS (%) spectra of the powders are shown in Figure 10a vs. the wavelength. All samples investigated display a characteristic absorption near 400 nm. The sharp rise in DRS at this wavelength is attributed to the ZnO band edge absorption. With Dy or Y doping, a systematic redshift of the absorption edge is observed. Therefore, to estimate the band gap energy ($E_{g}$) values, the Tauc relation is used, as illustrated in the equation below [44]

(2) $$\alpha h\upsilon =A{\left(h\upsilon -E_{g}\right)}^{n}$$

where hν represents the photon energy of the incident wavelength and A is an energy-independent constant, while n equals 2 or 3 for indirect (allowed or forbidden) transitions and 1/2 or 3/2 for direct (allowed or forbidden) transitions. F(R)  denotes the Kubelka–Munk (K-M) function, given by [44]

(3) $$F\left(R\right)=\frac{{\left(1-R\right)}^{2}}{2R}=\frac{\alpha }{S}$$

In this relation, R is the reflectance, α is the absorption coefficient, and S is the scattering coefficient.

The band gap energy ($E_{g}$) can be estimated by extending the linear portion of the graph plotting [F(R) hν]^2^ against hν. As we illustrate in Figure 10b–d, the bandgap systematically decreases with increasing Dy and Y concentrations. Pure ZnO exhibits a bandgap of 3.274 eV, while it decreases to 3.127 eV for ZY4 (4% Y) and further to 3.095 eV for ZD4 (4% Dy). This trend highlights the impact of the rare-earth dopant-induced structural modifications on ZnO’s electronic properties, which is supported by previous studies [13,60].

#### 3.5.2. PL Spectroscopy

**Figure 11:**
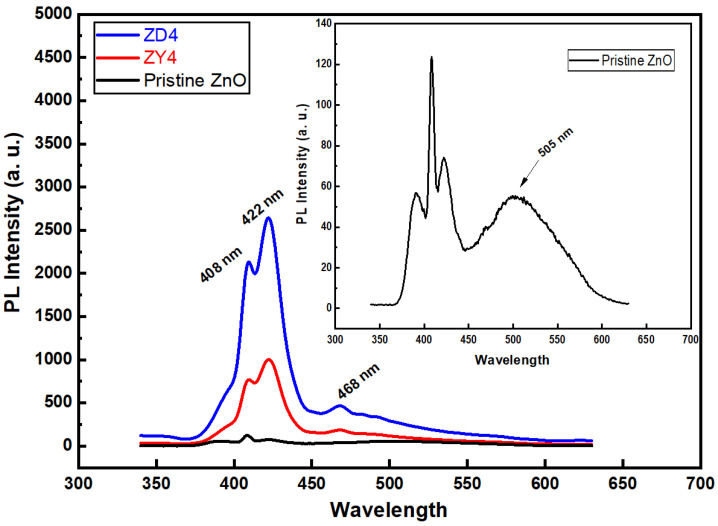
PL spectra of Dy/Y-doped ZnO samples with intensity variations linked to the dopant ratio.

Following the direct chemical evidence of doping-induced oxygen vacancy formation provided by XPS, we used photoluminescence (PL) spectroscopy to investigate the corresponding changes in the optical and electronic defect landscape of the ZnO nanoparticles after trivalent RE doping. Figure 11 shows the room-temperature PL spectra recorded under 325 nm of excitation, with emissions in the UV–visible range. The ZnO spectrum reveals a multi-component emission profile, including: a peak at 391 nm, a sharp, intense peak at 408 nm, a less intense peak at 422 nm, and a broad visible band centered at 505 nm. Such behavior is common for nanostructured ZnO. It arises from near-band–edge transitions and from radiative recombination through intrinsic deep-level defects [61,62]. After doping, the emission profile changes significantly. The native defect-related features become dominated by dopant-modified emissions. Their PL intensity increases by over an order of magnitude. The sharp peaks that we observe at 408 and 422 nm shift to approximately 409 and 423 nm, respectively. A new, distinct emission emerges at about 468 nm. Overall, the integrated PL intensity follows the order ZD4 > ZY4 >> ZnO. This shows that Dy^3+^ incorporation is more effective at generating radiative recombination centers. The pronounced effect of Dy^3+^ is attributed to its 4f^9^ electronic configuration, which introduces unique lattice perturbations and charge-compensation pathways that promote the formation of optically active centers [63,64]. The PL intensity hierarchy directly correlates with the oxygen vacancy concentration determined by XPS, confirming that the enhanced PL emission arises from increased defect states, particularly oxygen vacancies, which serve as radiative recombination centers.

### 3.6. Gas Sensor Efficiency

#### 3.6.1. Effect of Operating Temperature on Gas Sensing Performance

We systematically evaluate the gas sensing performance of the pristine, Dy-doped (ZD4), and Y-doped (ZY4) ZnO sensors toward three target gases: oxidizing nitrogen dioxide (NO_2_) and the reducing volatile organic compounds (VOCs) ethanol and acetone. We define the sensors’ response as $S=R_{gas}/R_{air}$ for oxidizing NO_2_ and $S=R_{air}/R_{gas}$ for reducing VOCs, where $R_{air}$ and $R_{gas}$ represent, respectively, the sensors’ resistance in air and the target gas. All measurements are conducted in a Teflon chamber under humid air at 50% RH and a flow rate of 1000 mL/min.

**Figure 12:**
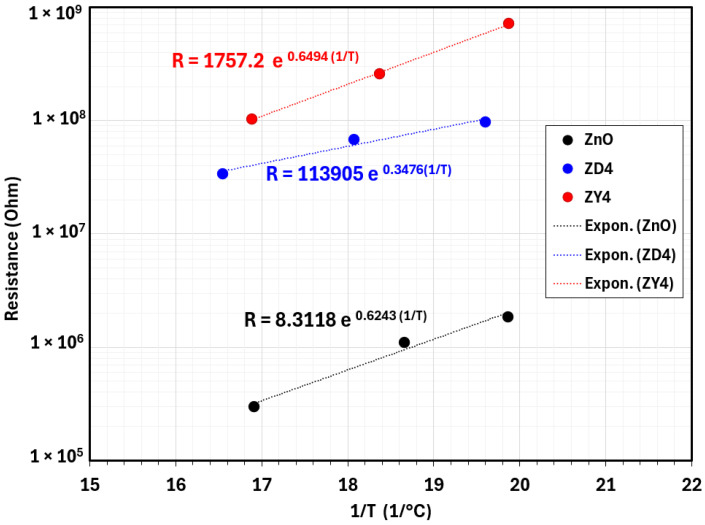
Arrhenius plots (ln R vs. 1000/T) of pristine ZnO, Dy-doped ZnO (ZD4), and Y-doped ZnO (ZY4) sensors, showing the linear fit used to extract the activation energies for electrical conduction.

OT is a key parameter that influences the sensing performance of the MOS gas sensors, as it directly affects gas adsorption/desorption processes and surface reaction kinetics. A preliminary temperature scan in humid air across the thermal range, going from 200 to 350 °C, was initially evaluated. As shown in Figure 12, the resistance of all three studied sensors exhibits a characteristic exponential decrease with increasing OT, thereby enhancing electrical conductivity by increasing the intrinsic carrier concentration. The RE-doped sensors present considerably higher baseline resistances compared with the pure ZnO-based sensor at any given temperature. This is consistent with the defect-rich structure induced by doping, as discussed. The temperature dependence can be expressed, therefore, according to the Arrhenius equation [65]:

(4) $$R=R_{0}\exp\left(\frac{E_{a}}{k_{B}T}\right)$$

Herein, $E_{a}$ is the activation energy for conduction, $k_{B}$ is the Boltzmann constant, and T is the absolute temperature.

By using the linear fit of  ln(R) vs. 1000/T , we calculate the activation energies; they are 0.054 eV for ZnO, 0.030 eV for ZD4, and 0.056 eV for ZY4. The lower activation energy of ZD4 indicates a higher density of shallow defect states, predominantly OVs, that facilitate carrier excitation at lower thermal energies. In contrast, the slightly higher activation energies of ZY4 and pristine ZnO suggest a more moderate defect landscape. Despite the low activation energies, the baseline resistance of the RE-doped sensors is approximately two orders of magnitude higher than that of pristine ZnO. This is attributed to the combined effect of (*i*) reduced carrier mobility due to scattering by oxygen vacancies and RE^3+^ substitutional defects, which act as potential barriers at grain boundaries; and (*ii*) reduced free carrier concentration, as the substitution of Zn^2+^ with RE^3+^ introduces trap states that localize electrons. Thus, while the small activation energy reflects low barriers for localized hopping, the overall transport is limited by both lower carrier density and reduced mobility.

**Figure 13:**
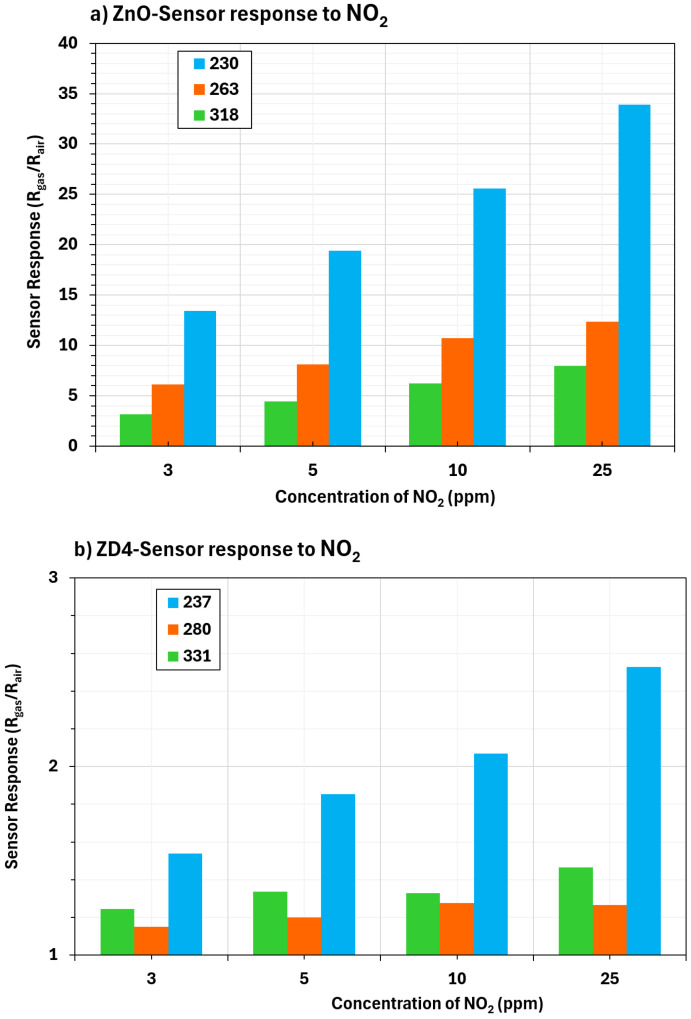

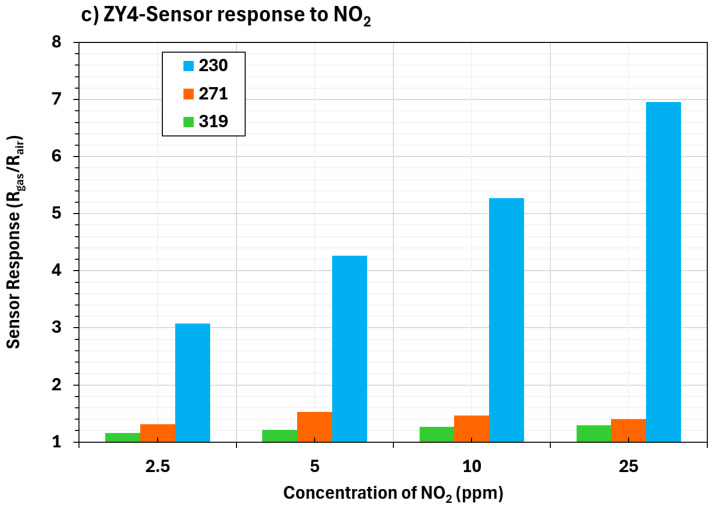
Effect of OT on the NO_2_ sensing performance of (**a**) pristine ZnO, (**b**) Dy-doped ZnO (ZD4), and (**c**) Y-doped ZnO (ZY4) sensors.

**Figure 14:**
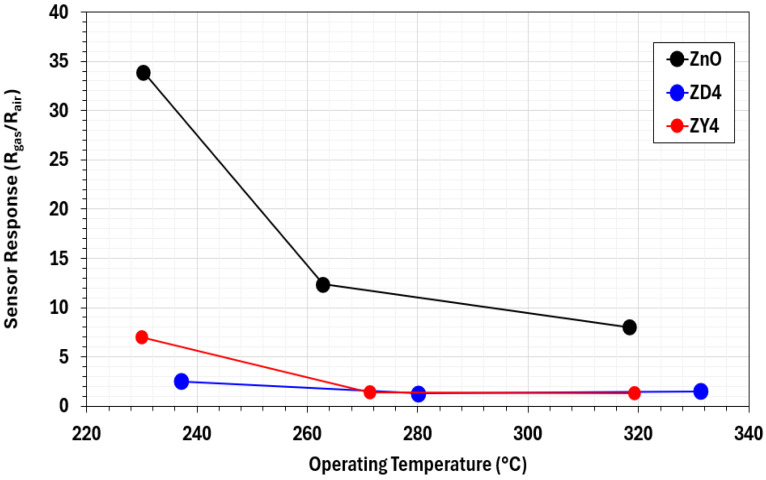
Sensors’ response to 25 ppm NO_2_ as a function of OT for pristine ZnO, Dy-doped ZnO (ZD4), and Y-doped ZnO (ZY4) sensors.

In order to identify the optimal OT for each sensor, we systematically evaluated the gas sensing performance under controlled gas exposure. We first measured NO_2_ sensing as a function of OT (2.5–25 ppm, Figure 13a–c). All sensors showed a concentration-dependent NO_2_ response, typical for n-type ZnO [66,67]. However, a pronounced temperature dependence was clearly demonstrated. For each material, the response dropped with increasing OT. This indicates that NO_2_ adsorption is thermodynamically favored at lower temperatures. Notably, pristine ZnO (Figure 13a) consistently exhibited the highest sensitivity to NO_2_ across the entire scanned temperature range (230–318 °C), while RE doping dramatically suppressed the NO_2_ response (Figure 13b,c). We then fixed NO_2_ at 25 ppm to find the optimal OT (Figure 14). Pure ZnO displayed a maximum response of 33.88 at 230 °C, while ZD4 and ZY4 reached, respectively, their highest responses of 2.53 at 237 °C and 6.96 at 230 °C. These results confirm that RE doping significantly attenuates the electron-withdrawing interaction between NO_2_ and the ZnO surface.

**Figure 15:**
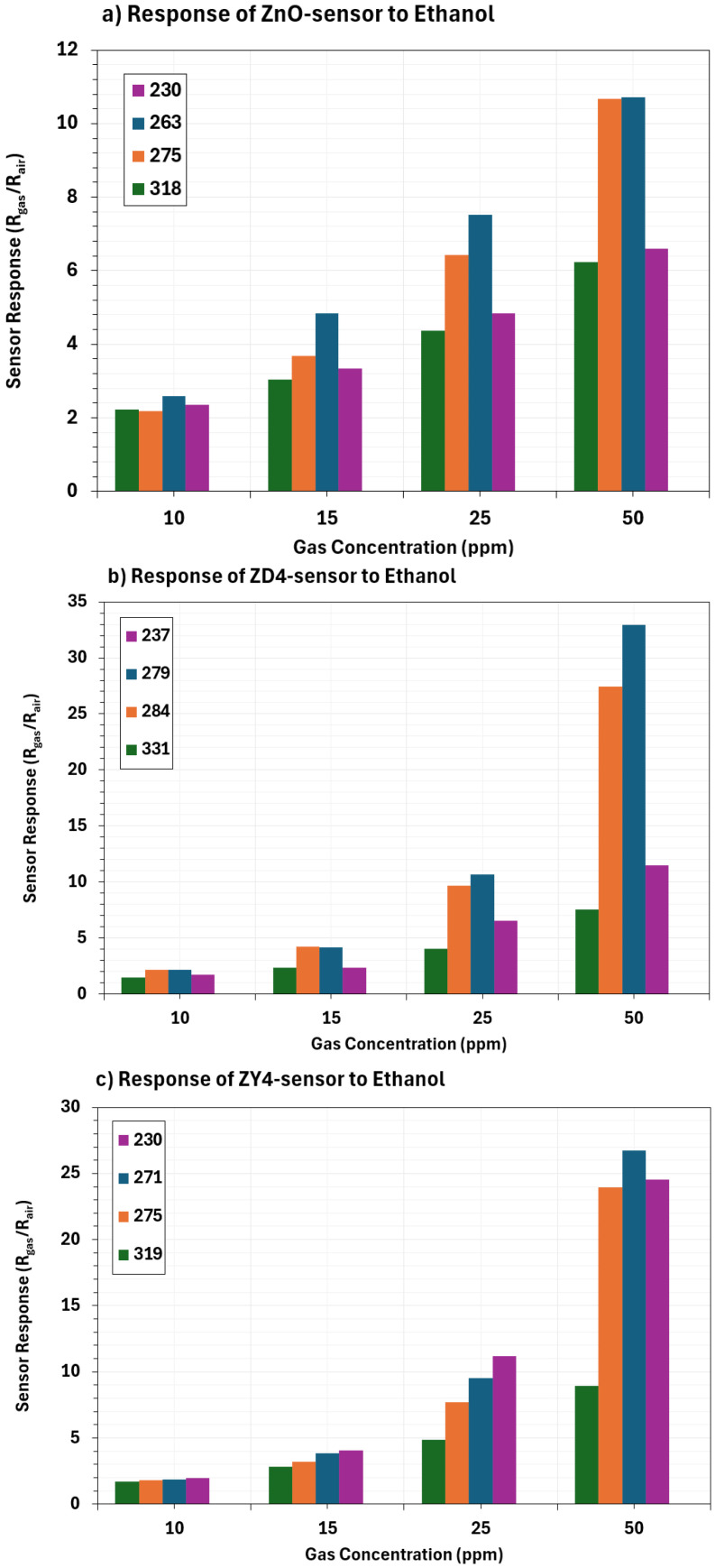
Effect of OT on the ethanol sensing performance of (**a**) pristine ZnO, (**b**) Dy-doped ZnO (ZD4), and (**c**) Y-doped ZnO (ZY4) sensors.

Similarly, ethanol sensing performance was evaluated from 10 to 50 ppm at various OTs ranging from 230 to 318 °C. As shown in Figure 15a–c, all sensors exhibited a typical reducing gas behavior, with the response increasing monotonically with concentration; i.e., ethanol molecules react with surface chemisorbed oxygen species, releasing electrons into the CB and reducing the resistance. Consequently, higher ethanol concentrations produce stronger surface reactions and larger response values [68,69].

Unlike NO_2_, ethanol sensing displays a clear maximum at intermediate temperatures. It reflects the balance between thermally activated surface reactions and gas desorption. For this gas, pristine ZnO shows an optimal temperature of 263 °C. The influence of OT is more pronounced for the RE-doped sensors than for pure ZnO. Specifically, doping shifts the optimum to higher temperatures (279 °C for ZD4 and 271 °C for ZY4). For example, at 50 ppm, ZD4 achieved a response of 32.91 at 279 °C, approximately three times higher than the pure sample detector.

**Figure 16:**
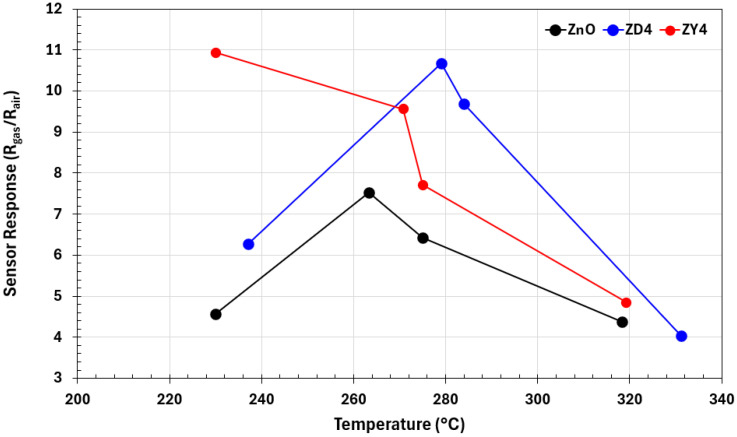
Sensors’ response to 25 ppm ethanol as a function of OT for pristine ZnO, Dy-doped ZnO (ZD4), and Y-doped ZnO (ZY4) sensors.

To clearly identify the optimum OT for ethanol detection and to allow a direct comparison among the three sensors, we plotted the responses vs. temperature toward a fixed ethanol concentration selected at 25 ppm. As shown in Figure 16, the pure ZnO sensor demonstrates a maximum response of 7.52 at 263 °C. Meanwhile, ZD4 and ZY4 reach their highest response of 10.67 and 10.94 at, respectively, 279 °C and 230 °C. These results confirm that ethanol sensing is optimized at intermediate temperatures, with the exact optimum depending on the dopant type.

**Figure 17:**
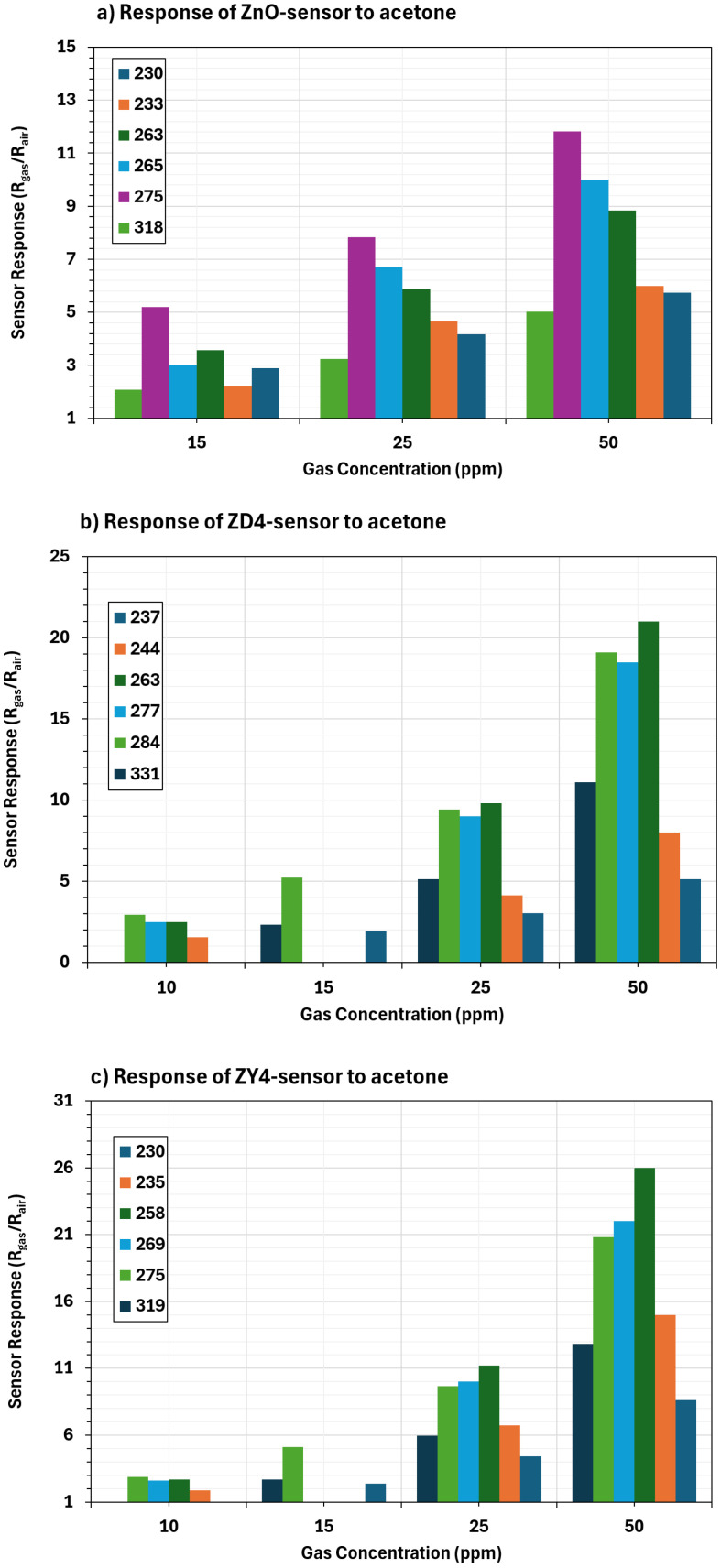
Effect of OT on the acetone sensing performance of (**a**) pristine ZnO, (**b**) Dy-doped ZnO (ZD4), and (**c**) Y-doped ZnO (ZY4) sensors.

**Figure 18:**
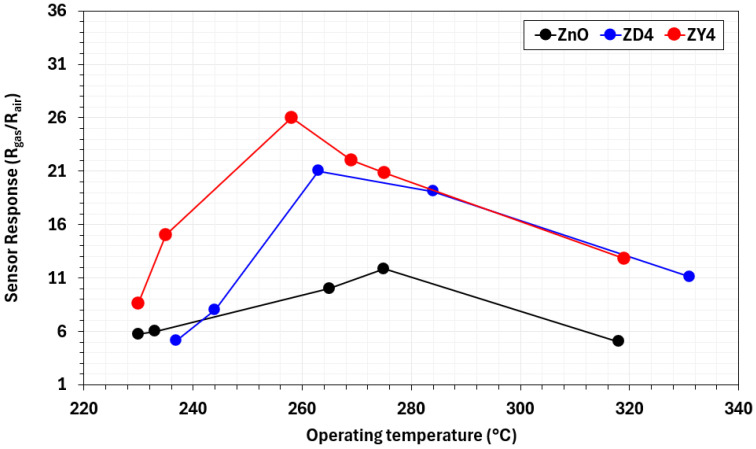
Sensors’ response to 50 ppm acetone as a function of OT for pristine ZnO, Dy-doped ZnO (ZD4), and Y-doped ZnO (ZY4) sensors.

Acetone sensing followed similar trends to ethanol, with concentration-dependent responses (10–50 ppm) and an optimal temperature. Figure 17 shows that the response for all sensors increases with the acetone concentration, confirming that higher gas concentrations produce larger response signals and, consequently, stronger surface reactions. As observed, acetone sensing exhibits a pronounced dependence on the OT, with a clear maximum at intermediate temperatures for all samples. The optimal temperature for pristine ZnO is measured at approximately 275 °C. However, a stronger temperature dependence is observed for the RE-doped sensors, as demonstrated in Figure 18 for a fixed acetone concentration selected at 50 ppm. For ZD4, the acetone response rises sharply with temperature and attains a highest value of 21 at 263 °C, followed by slightly lower but still significant responses at nearby temperatures. Similarly, ZY4 exhibits the highest acetone response among all samples, reaching a maximum of 26 at 258 °C, followed by a gradual decrease at higher temperatures.

#### 3.6.2. Dynamic Gas Response Behavior and Sensing Kinetics

**Figure 19:**
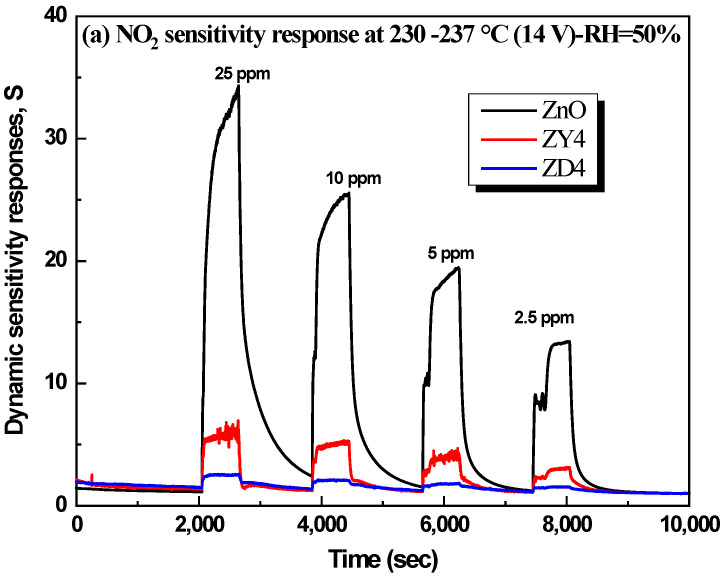

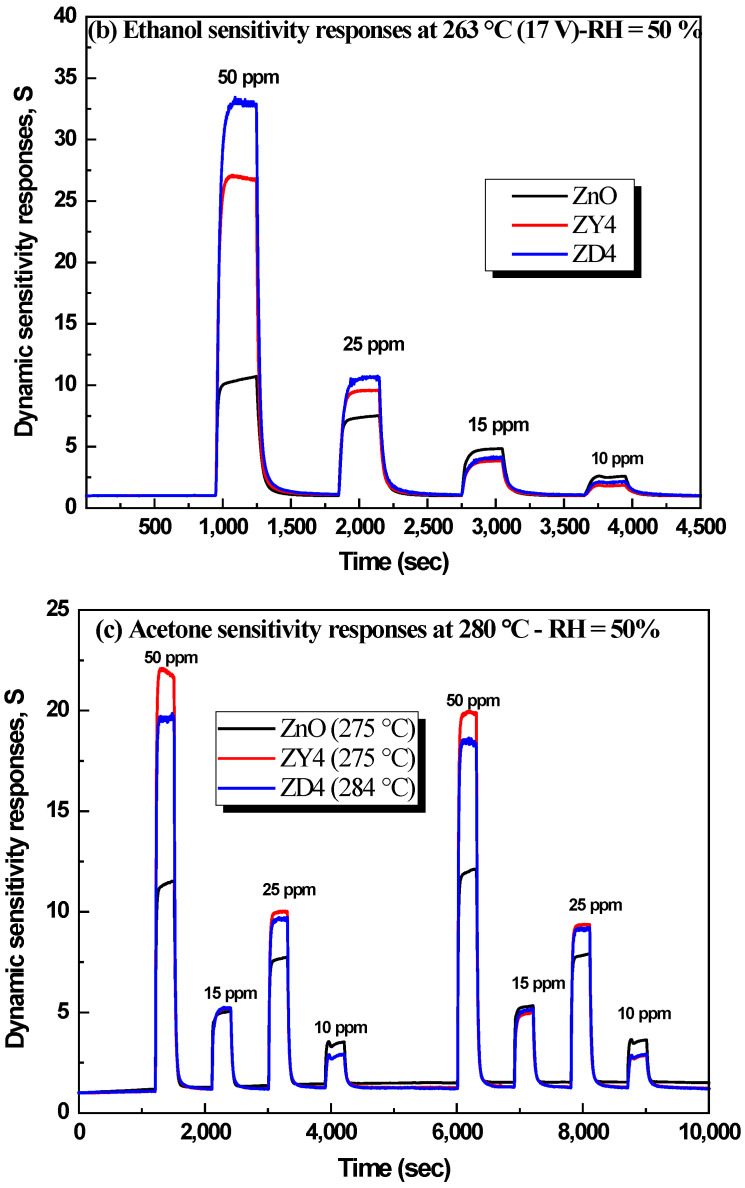
Dynamic sensitivity responses of ZnO, Y-doped ZnO (ZY4), and Dy-doped ZnO (ZD4) sensors toward (**a**) NO_2_ at 230–237 °C (14 V, RH = 50%), (**b**) ethanol at 263 °C (17 V, RH = 50%), and (**c**) acetone at 280 °C (RH = 50%).

Figure 19 presents the dynamic sensing responses (S vs. time) of the pure and the doped ZnO sensors toward NO_2_, ethanol, and acetone at their respective optimal OTs. We note that all sensors show stable baselines and reproducible response–recovery cycles, confirming the good operational stability of the sensors. For NO_2_ (Figure 19a), pristine ZnO shows the strongest and the most concentration-dependent response, while ZY4’s and ZD4’s responses are significantly suppressed. Conversely, for ethanol and acetone (Figure 19b,c), the doped sensors displayed markedly enhanced responses compared with the pure sample, with ZD4 showing the most marked step response at higher VOC concentrations.

**Figure 20:**
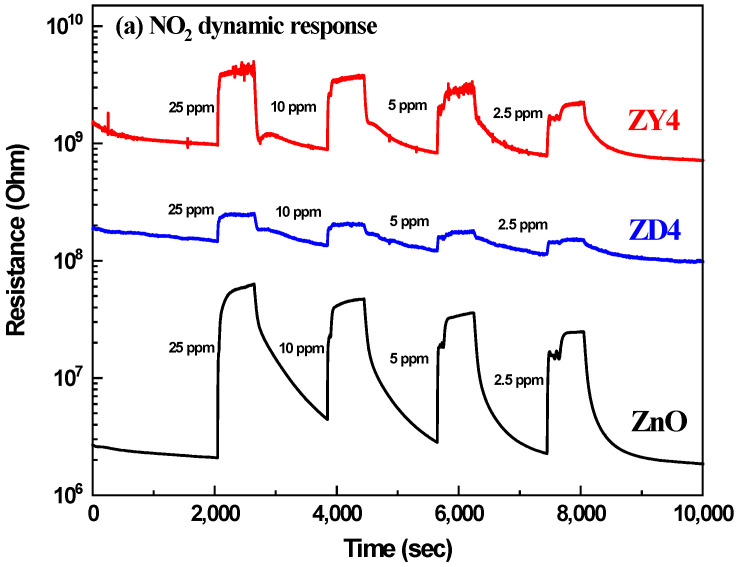

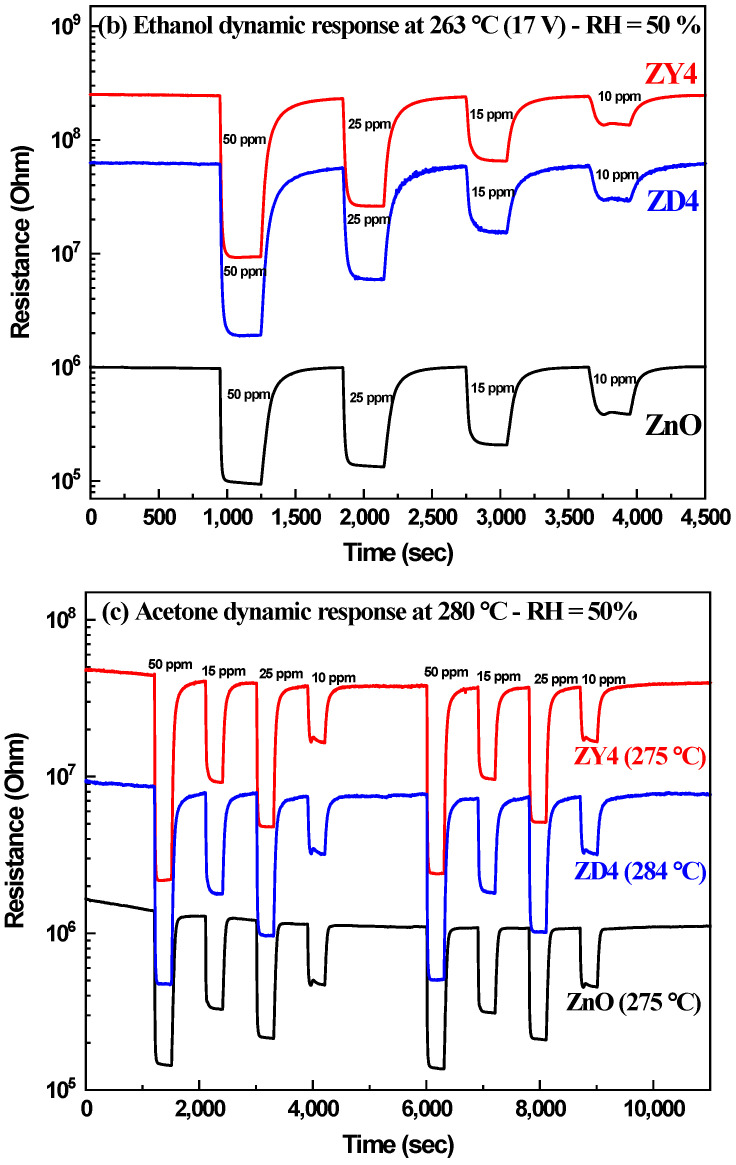
Dynamic resistance–time curves of ZnO, Y-doped ZnO (ZY4), and Dy-doped ZnO (ZD4) sensors under exposure to (**a**) NO_2_, (**b**) ethanol at 263 °C (17 V, RH = 50%), and (**c**) acetone at 280 °C (RH = 50%).

We present the corresponding dynamic resistance–time curves in Figure 20. Under NO_2_ exposure, ZnO displays substantial resistance increases (consistent with the strong electron withdrawal), while the RE-doped samples show minimal resistance variation. Meanwhile, under VOC exposure, doped sensors exhibited faster and larger resistance decreases, indicating more efficient charge transfer during surface redox reactions.

#### 3.6.3. Quantitative Performance Evaluation and Mechanisms

**Figure 21:**
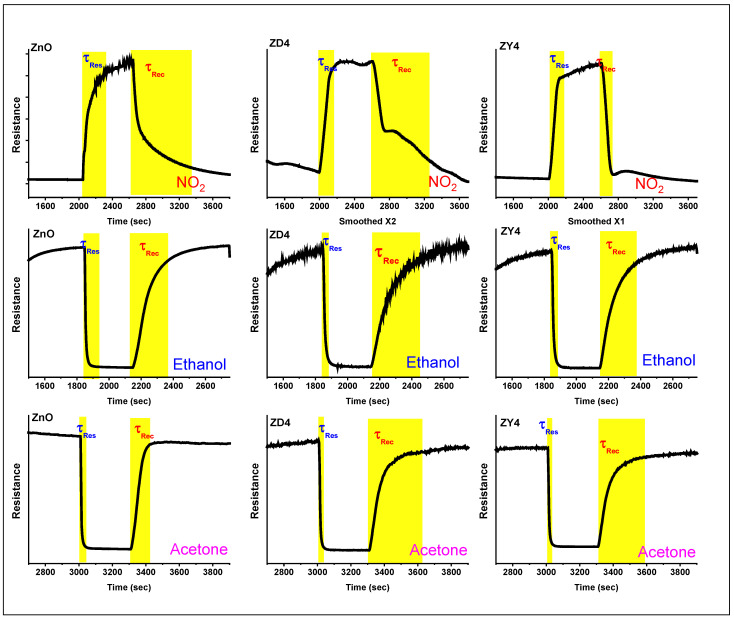
Dynamic resistance–time curves of pristine ZnO, Dy-doped ZnO (ZD4), and Y-doped ZnO (ZY4) sensors recorded at 25 ppm for (**top** row) NO_2_, (**middle** row) ethanol, and (**bottom** row) acetone at their respective optimum OTs. The shaded regions indicate the response time (τ_Res_) and recovery time (τ_Rec_).

Figure 21 defines response time (τ_Res_) and recovery time (τ_Rec_) (90% of total change). In Table 4, we summarize the extracted data. They confirm a dopant-induced selectivity inversion: ZD4 is ethanol-selective (5.6× over NO_2_), and ZY4 is acetone-selective (2× over NO_2_).

In air, oxygen molecules adsorb and extract conduction-band electrons to form chemisorbed ionic species according to the following equations [70]:

(5) $$O_{2}\;\left(\mathrm{gas}\right)\;\to \;O_{2}\;\left(\mathrm{ads}\right)\;$$

(6) $$O_{2}\;\left(\mathrm{ads}\right)+e^{-}\to \;O_{2}^{-}\left(\mathrm{ads}\right)$$

(7) $$O_{2}^{-}\left(\mathrm{ads}\right)+e^{-}\;\to \;2O^{-}\left(\mathrm{ads}\right)$$

This mechanism produces a surface electron-depletion layer while increasing the baseline resistance. Substituting the smaller Zn^2+^ with these larger RE cations creates a local charge imbalance. The system compensates for this imbalance primarily by generating oxygen vacancies ($V_{O}^{..}$), a crucial process by the defect reaction described in Equation (8)

(8) $$2{\mathrm{RE}}_{2}O_{3}\overset{\mathrm{ZnO}}{\to }4{\mathrm{RE}}_{\mathrm{Zn}}^{.}+V_{O}^{..}+6O_{O}^{x}$$

where ${\mathrm{RE}}_{\mathrm{Zn}}^{.}$ represents the trivalent dopant at the zinc sites (effective positive charge) and $O_{O}^{x}$ is the lattice oxygen. The gas response profile is governed by the engineered vacancy-rich surface, which operates through two distinct pathways depending on the target gas itself. These vacancies act as electron donors, thereby reducing the net electron transfer to the adsorbed, electron-withdrawing NO_2_ molecules according to Equations (9) and (10).

(9) $${\mathrm{NO}}_{2}(\mathrm{gas})+e^{-}\to {\mathrm{NO}}_{2}^{-}(\mathrm{ads})\;$$

(10) $${\mathrm{NO}}_{2}(\mathrm{gas})+O^{-}(\mathrm{ads})+e^{-}\to {\mathrm{NO}}_{3}^{-}(\mathrm{ads})$$

The significantly suppressed NO_2_ response on RE-doped surfaces can be further understood by considering the adsorption configuration and electronic interactions. On ZnO, NO_2_ acts as a strong electron acceptor, extracting electrons from the surface and forming NO_2_^−^ species (Equation (9)). The presence of OVs on RE-doped surfaces introduces localized donor states near the CB minimum. These donor states trap electrons, effectively reducing the driving force for electron transfer from the surface to the NO_2_ molecule. This is consistent with the observed increase in baseline resistance, which indicates that electrons are partially localized at defect states rather than being freely available for NO_2_ adsorption.

Furthermore, the substitution of Zn^2+^ with trivalent RE^3+^ ions introduces a net positive charge at the cation sites, which can alter the surface’s acid–base properties. The RE^3+^ ions, particularly Dy^3+^ with its partially filled 4f^9^ configuration, act as Lewis acid sites that preferentially interact with electron-rich molecules. This modification of surface acidity weakens the interaction with NO_2_ (a relatively weak Lewis base) while enhancing the adsorption of VOCs such as ethanol and acetone, which can act as stronger Lewis bases through their oxygen-containing functional groups.

Equation (8) represents the defect reaction for trivalent RE^3+^ substitution in ZnO. This reaction is thermodynamically driven by the large formation energy of oxygen vacancies in ZnO, which is compensated by the gain in lattice energy from substituting Zn^2+^ with RE^3+^. The formation of one oxygen vacancy compensates for the charge of two substitutional RE^3+^ defects, maintaining overall charge neutrality. The binding energy of the RE-O bond in the oxide matrix (approximately 5.5–6.5 eV for RE-O) further stabilizes the substitutional configuration.

Regarding the VOC oxidation reactions, it should be noted that ethanol and acetone oxidation on the hydroxylated ZnO surface are more complex than simple reactions with O^−^ species. In the presence of surface hydroxyl groups (confirmed by XPS and FTIR analyses), the oxidation of VOCs proceeds through surface-mediated reactions involving both oxygen species and hydroxyl groups:

C_2_H_5_OH(ads) + O^−^(ads) + OH^−^(ads) → CH_3_CHO(ads) + H_2_O(ads) + 2e^−^ → CO_2_ + H_2_O (11)

CH_3_COCH_3_ (ads) + 2O^−^(ads) + 2OH^−^(ads) → 3CO_2_ + 3 H_2_O + 8e^−^ (12)

These reactions are facilitated by the abundant surface hydroxyl groups present on the RE-doped ZnO surfaces, as confirmed by the XPS and FTIR analyses.

The strong correlation between the oxygen vacancy concentration and suppressed NO_2_ response observed in this study is consistent with recent findings on defect-engineered ZnO sensors. Ciftyurek et al. demonstrated that oxygen vacancies act as surface active sites that localize electrons [71], thereby reducing the driving force for electron transfer from the ZnO surface to electron-withdrawing NO_2_ molecules [31,72]. This mechanism is further supported by DFT calculations on rare-earth-doped ZnO heterojunctions, which revealed that vacancy-induced donor states weaken NO_2_ adsorption energy and consequently suppress the gas response [73].

When doping ZnO with Dy^3+^ ions, the partially filled 4f^9^ shell generates localized states. This process, in turn, imparts a distinct Lewis acid character to the surface. Accordingly, the pronounced ethanol selectivity arises from preferential adsorption through the hydroxyl group [74], consistent with recent studies on defect-rich ZnO surfaces, where oxygen vacancies promoted enhanced interaction with polar molecules through hydroxyl-mediated surface reactions [75]. As for the Y^3+^ doping ion, the 4d^1^ orbital generates a different surface environment. This electronic structure fosters a surface environment conducive to the selective adsorption and activation of acetone and seems to weaken NO_2_ adsorption. The acetone-selective behavior of Y-doped ZnO is further supported by recent work on rare-earth-doped ZnO, where Y^3+^ substitution introduced surface states that favor the adsorption of ketone groups through Lewis acid–base interactions [74]. Hence, the Y-doped sensor exhibited the fastest recovery, as shown in Table 4, indicating a more transient, less favorable interaction with the target gases. To contextualize the performance of our sensors, Table 5 compares the gas sensing characteristics obtained in this work with verified literature data for rare-earth-doped ZnO sensors.

## 4. Conclusions

We tailored the structural and the electronic properties of ZnO nanoparticles through targeted doping with trivalent rare-earth ions, namely Y^3+^ and Dy^3+^, using a scalable solid-state synthesis route. Through XRD and XPS analysis, we confirmed the successful dopant incorporation that introduced measurable lattice strain and produced a well-defined hierarchy in the OV concentration (ZD4 > ZY4 > ZnO). While doping suppressed the NO_2_ response by up to 94%, it simultaneously enhanced sensitivity toward key VOCs. Notably, Dy^3+^ doping produces ethanol selectivity (5.6× over NO_2_), whereas Y^3+^ doping favors acetone (2× over NO_2_). Future work will extend this study through (i) systematic repeatability testing across a larger device batch to statistically validate the observed selectivity trends; (ii) long-term stability and humidity-dependence studies, currently ongoing; and (iii) evaluation under realistic multi-gas conditions.

## Figures and Tables

**Table 1:** Properties of gas sensors based on ZnO semiconductors doped with different rare-earth oxides.

RE Element	Gas Target	Detection Limit (ppm)	Operation Temperature (°C)	Ref.
**Ce**	Ethanol	100	310	[33]
		5	320	[34]
**Sm**	Ethanol	100	340	[35]
	NH_3_	25	RT	[36]
**La**	H_2_	10	300	[37]
	NH_3_	0.5	RT	[38]
	Formaldehyde (HCHO)	1	250	[39]
	Acetone	10	330	[40]
**Nd**	NH_3_	25	RT	[36]
**Dy**	NO_2_	1	150	[22]
	Ethanol	50	300	[29]
**Y**	Gasoline	10	200	[25]
	Ethanol	10	400	[25]

**Table 2:** Refined unit cell parameters for ZnO, ZY4, and ZD4.

	a (Å)	Err	c (Å)	Err	ϑ (Å^3^)	Err	R_p_ (%)	R_wp_ (%)	χ^2^
**Pristine ZnO**	3.2499	0.0002	5.2064	0.0003	47.621	0.005	-	-	-
**ZY4**	3.2514	0.0002	5.2084	0.0002	47.684	0.005	3.2	4.8	1.9
**ZD4**	3.2510	0.0002	5.2072	0.0004	47.661	0.006	3.2	4.8	1.9

**Table 3:** The strain and the crystallite size of the prepared samples.

Samples	Crystallite Size (nm)	Strain
**Pristine**	7.7	0.0166 (4)
**ZY4**	9.5	0.13 (2)
**ZD4**	21.2	0.17 (1)

Note: Numbers in parentheses indicate the standard error of the fit in the last digit (e.g., 0.0166 (4) means 0.0166 ± 0.0004).

**Table 4:** Quantitative gas sensing parameters of ZnO, Y-doped ZnO (ZY4), and Dy-doped ZnO (ZD4) sensors toward NO_2_, ethanol, and acetone at a selected concentration of 25 ppm and their respective optimum OTs. Values represent representative measurements selected from three fabricated devices per composition (see Section 2.2.2).

Gas	Sensor	R_air_ (Ω)	Response (S)	τ_Res_ (s)	τ_Rec_ (s)	τ_Rec_/τ_Res_
**NO**_2_	ZnO	2.09 × 10^6^	30.0	343.40	705.68	2.06
	ZD4	1.475 × 10^8^	1.71	157.47	609.01	3.87
	ZY4	1.027 × 10^9^	4.17	208.93	99.75	0.48
**Ethanol**	ZnO	9.964 × 10^5^	7.47	18.89	224.81	11.90
	ZD4	5.713 × 10^7^	9.61	30.43	284.67	9.36
	ZY4	2.315 × 10^8^	8.84	24.17	228.98	9.47
**Acetone**	ZnO	1.207 × 10^6^	5.67	12.19	100.68	8.26
	ZD4	7.905 × 10^6^	8.23	18.29	350.75	19.17
	ZY4	3.948 × 10^7^	8.27	12.19	271.45	22.27

**Table 5:** Comparison of the gas sensing performance of ZnO-based sensors from the literature and this work.

Sensor Material	Target Gas	Response (S)	Conc. (ppm)	Temp (°C)	Response Time (s)	Recovery Time (s)	Ref.
**ZnO**	NO_2_	30.0	25	230	343	706	This work
**Dy-doped ZnO**	NO_2_	1.71	25	237	157	609	This work
**Y-doped ZnO**	NO_2_	4.17	25	230	209	100	This work
**ZnO**	Ethanol	7.47	25	263	19	225	This work
**Dy-doped ZnO**	Ethanol	9.61	25	279	30	285	This work
**Y-doped ZnO**	Ethanol	8.84	25	271	24	229	This work
**ZnO**	Acetone	5.67	25	275	12	101	This work
**Dy-doped ZnO**	Acetone	8.23	25	263	18	351	This work
**Y-doped ZnO**	Acetone	8.27	25	258	12	271	This work
**Y-doped ZnO nanorods (1 at%)**	Acetone	Higher than pure ZnO	100	—	~30	~90	[76]
**Ce-doped ZnO (6 mol%)**	Ethanol	~30	50	320	2	16	[34]
**Ce-doped ZnO (5 at%)**	Ethanol	~80	100	320	—	—	[34]
**Nd-doped ZnO (2 at%)**	Ethanol	37.8	100	—	—	—	[77]
**Sm-loaded ZnO (5 wt%)**	Ethanol	60× higher than pure ZnO	—	300	—	—	[78]
**La-doped ZnO (1.0 wt%)**	Acetone	64	200	340	—	—	[79]
**La-doped ZnO thin film**	Acetone	82.57%	50	150	26	27	[80]
**Gd-doped ZnO nanosheets**	Ethanol	11.8× higher than pure ZnO	—	—	—	—	[81]
**Tb-doped ZnO (4%)**	Ethanol/acetone	Maximum response	—	—	—	—	[82]

## Data Availability

The original contributions presented in this study are included in the article. Further inquiries can be directed to the corresponding author.
